# Effectiveness of Meningococcal C Conjugate Vaccine in Salvador, Brazil: A Case-Control Study

**DOI:** 10.1371/journal.pone.0123734

**Published:** 2015-04-13

**Authors:** Cristiane Wanderley Cardoso, Guilherme Sousa Ribeiro, Mitermayer Galvão Reis, Brendan Flannery, Joice Neves Reis

**Affiliations:** 1 Gonçalo Moniz Research Center, Oswaldo Cruz Foundation, Brazilian Ministry of Health, Salvador, Brazil; 2 Municipal Secretary of Health of Salvador, Salvador, Brazil; 3 Institute of Collective Health, Federal University of Bahia, Salvador, Brazil; 4 Pan American Health Organization, Brasília, Brazil; 5 School of Pharmacy, Federal University of Bahia, Salvador, Brazil; University of Cambridge, UNITED KINGDOM

## Abstract

**Background:**

During a citywide epidemic of serogroup C meningococcal disease in Salvador in 2010, Brazil, the state government initiated mass vaccination targeting two age groups with high attack rates: individuals aged <5 years and 10–24 years. More than 600,000 doses of meningococcal serogroup C conjugate vaccines were administered. We performed a case-control study to evaluate vaccine uptake, document vaccine effectiveness and identify reasons for non-vaccination.

**Methods and Findings:**

Population-based surveillance identified patients with laboratory-confirmed invasive meningococcal C (MenC) disease during 2010. Information on MenC vaccination was obtained from case patients and age-matched individuals from the same neighborhoods. MenC vaccine effectiveness was estimated based on the exact odds ratios obtained by conditional logistic regression analysis. Of 51 laboratory-confirmed cases of serogroup C meningococcal disease among patients <5 and 10–24 years of age 50 were included in the study and matched with 240 controls. Overall case-fatality was 25%. MenC vaccine coverage among controls increased from 7.1% to 70.2% after initiation of the vaccination campaign. None of the 50 case patients but 70 (29.2%) of the 240 control individuals, including 59 (70.2%) of 84 matched with cases from the period after MenC vaccination, had received at least one MenC vaccine dose. Overall effectiveness of MenC was 98% with a lower 95% exact confidence limit of 89%.

**Conclusions:**

MenC vaccines administered during the meningococcal epidemic were highly effective, suggesting that rapid vaccine uptake through campaigns contributed to control of meningococcal disease.

## Introduction

*Neisseria meningitidis* causes severe and life-threatening invasive diseases, such as meningitis and meningococcal sepsis. Despite timely antibiotic treatment, case-fatality often exceeds 10% and one quarter of survivors develop significant neurological disabilities or require limb amputation [1]. Meningococcal disease affects individuals of all age groups, but incidence is highest among children under five years of age. During epidemics and in outbreak situations, meningococcal disease incidence tends to increase mainly among older children, adolescents and young adults [2].

An epidemic of serogroup C meningococcal disease occurred in 2010 in the city of Salvador, the state capital and third most populous city of Brazil (estimated population 2.7 million, in 2010). To combat the epidemic, the state government introduced a conjugate vaccine against serogroup C meningococcal (MenC) disease for children <5 years prior to national introduction of MenC vaccination in Brazil’s National Immunization Program. However, incidence of meningococcal disease continued to increase among older children and adolescents. Therefore, the MenC vaccination campaign was gradually extended to also include those 10–24 years of age. In total, more than 611,673 doses of MenC vaccine were administered during the campaigns with estimated coverage of 92% among the target population of children aged <5 years, 80% among 10–14 year olds, 67% among 15–19 year olds and 41% among adults aged 20–24 years [3]. Mass vaccination of residents of Salvador was associated with decreased MenC incidence in targeted age groups [3]. Although MenC cases continued to occur, no confirmed cases of serogroup C meningococcal disease were reported among vaccinated individuals.

In order to estimate the effectiveness of MenC vaccination in containing this citywide epidemic of serogroup C meningococcal disease and to collect information on uptake of MenC vaccine among targeted age groups and on reasons for non-vaccination in areas in which cases occurred, we conducted a case-control study of confirmed cases of serogroup C meningococcal disease identified before and after mass vaccination.

## Materials and Methods

### Mass vaccination against meningococcal serogroup C disease

MenC vaccine was introduced in four stages: vaccination of children <5 years of age began in February, 2010; followed by mass vaccination of children 10–14 years of age on 30–31 May; adolescents 15–19 years of age on 12–13 June; and adults 20–24 years on 14–15 August, 2010. Children <12 months received two doses of MenC vaccine in the first six months of life followed by a third dose at 12–15 months of age; all other age groups received just one vaccine dose, including those 12–59 months of age in February 2010. Initially, MenC-tetanus toxoid conjugate vaccine (MenC-TT, Neisvac, Baxter vaccines) was used in the vaccination campaigns, being replaced by MenC-CRM197 conjugate vaccine (Novartis vaccines) in August, 2010 [3].

### Surveillance for meningococcal disease cases

Cases of meningococcal disease were identified between January 1^st^ and December 31^st^ of 2010 as part of ongoing, population-based surveillance in Salvador, Brazil [4]. Reporting of suspected cases of meningococcal disease to health authorities is mandatory in Brazil. During 2010, the state reference hospital for infectious diseases (Couto Maia Hospital) reported 86% of the suspected cases of meningococcal disease in the city of Salvador, Brazil. We used two methods to ascertain meningococcal disease cases. First, we performed active hospital-based surveillance at Couto Maia Hospital by reviewing laboratory records five days a week to identify patients with evidence of *N*. *meningitidis* infection in blood or cerebrospinal fluid. In addition, we assessed the national Notifiable Diseases Information System [*Sistema de Informação de Agravos de Notificação* (SINAN)] to identify cases of meningococcal disease from other public or private health facilities in Salvador.

We included in this case-control study all cases of laboratory confirmed serogroup C meningococcal disease among residents of Salvador, Brazil in age groups targeted for MenC vaccination (<5 years of age or 10–24 years of age). A laboratory-confirmed case of meningococcal disease was defined as isolation of *N*. *meningitidis* from blood or cerebrospinal fluid (CSF) from a patient with at least three clinical signs or symptoms of meningococcal disease (fever, headache, vomiting, neck stiffness, meningeal irritation, seizures, petechial or purpural rash). Patients with laboratory-confirmed *N*. *meningitidis* disease caused by a non-C serogroup and those for whom serogroup were not determined were excluded from the case-control study. Patients with clinical signs and symptoms compatible with meningococcal disease without laboratory confirmation were not included.

### *N*. *meningitidis* isolation and serogrouping

*N*. *meningitidis* isolated from patients with meningococcal disease were sent to the Central Public Health Laboratory for the state of Bahia and/or to the Molecular Biology and Pathology Laboratory at the Gonçalo Moniz Research Center, Oswaldo Cruz Foundation, in Salvador for characterization using serogroup-specific antisera (Difco Laboratories, Detroit, MI, USA) [4,5].

### Control selection

For each case we attempted to select four or eight community controls, matched with cases by age and neighborhood of residence. Age-matching was performed according to targeted age groups for vaccination: <5, 10–14, 15–19 and 20–24 years of age. Four controls were matched with cases that occurred before MenC vaccination campaigns for each targeted age group, while eight controls were matched for cases that occurred after MenC vaccination campaigns. We increased the number of control individuals for the period after MenC vaccination campaigns because we expected fewer cases and higher vaccination coverage among controls in that period. To identify four eligible control individuals in the same age group as case-patients with illness onset before vaccination campaigns, study teams searched residences or apartments beginning with the residence immediately to the left of the case-patient household and continuing to visit each of the first four residences to the left, then each of the first four residences to the right, then the residence facing the case-patient household, and each of the four residences to the left and right of that residence. To identify eight eligible control individuals for case-patients with illness onset after vaccination campaigns, the same strategy was used except that each of the first eight houses was approached in each direction. Searches in multi-story buildings continued on neighboring floors. Eligible individuals for whom consent was not obtained were replaced. Study teams continued searching for eligible individuals until they had enrolled the pre-determined number of control individuals for each case-patient.

### Data collection

For the cases identified during active case finding at Couto Maia Hospital, we collected data on demographics, clinical presentation and laboratory findings by interviewing the patient or legal guardian and by medical chart review using standardized data entry forms. For cases identified from the SINAN database, we collected data by interview to complete the information obtained from SINAN. Data were collected by proxy from family members of deceased case patients. Demographic data were also collected from controls using the standardized questionnaire. For both case-patients and control individuals, study teams asked persons aged 10–24 years about student status or work outside the household (for those aged 18 years and older), number of people living in the household, number of rooms in the household and whether there were any current smokers in the household. Data on prior use of MenC vaccination, on number of MenC vaccine doses received and date of vaccination were collected through review of personal vaccination cards, the official document used by the Brazilian Ministry of Health to record vaccination history. If the vaccination card was unavailable, we collected data on MenC vaccination by interview of the study subject, proxy or legal guardian, to obtain verbal information on vaccine use. Self-report of MenC vaccination was accepted if respondents could provide information on three of five questions about date, provider or injection site. This validation was adapted from the verbal validation used in Brazil during a national rubella vaccination campaign in 2008 [6]. Study participants were considered vaccinated if they had received one or more dose of MenC conjugate vaccine at least 14 days before the date of the corresponding case patient’s disease onset. Reasons for non-vaccination were collected for individuals in targeted age groups who were not vaccinated in MenC vaccination campaigns.

### Ethics Statement

The study was approved by the Institutional Review Board of the Centro de Pesquisa Gonçalo Moniz/FIOCRUZ (IORG00002090/IRB000026120). Written informed consent was obtained from all participants and/or their parents.

### Statistical analysis

Case and control data were double entered and validated in Epi-Info version 3.5.1 (CDC /USA). Clinical characteristics of cases were described by absolute and relative frequencies or by means and standard deviations. We used univariate conditional logistic regression to assess whether case and control individuals were similar in relation to known risk factors for meningococcal disease, including presence of smokers in the household and household crowding. We compared MenC conjugate vaccine coverage among controls from the period before and after vaccine introduction for all control individuals combined and stratified by age group, MenC vaccine source (public versus private) and documented versus self-reported vaccination. Exact odds ratios (OR) for MenC vaccination and 95% confidence intervals (95% CI) were estimated using exact conditional logistic regression analysis, which provides a point estimate and 95% confidence limit in the absence of cases among vaccinated persons. MenC vaccine effectiveness was calculated as (1—OR) x 100. Separate estimates of MenC vaccine effectiveness were calculated for the period before and after public vaccination, and considering only documented vaccine doses. Reasons for non-vaccination in MenC vaccination campaigns were contrasted for cases and controls using McNemar’s test. For all comparisons, statistical significance was set at *p*<0.05. Statistical analyses were performed in Epi Info version 3.5.1. or in SAS 9.3 (SAS Institute Inc.; USA).

## Results

**Fig 1:**
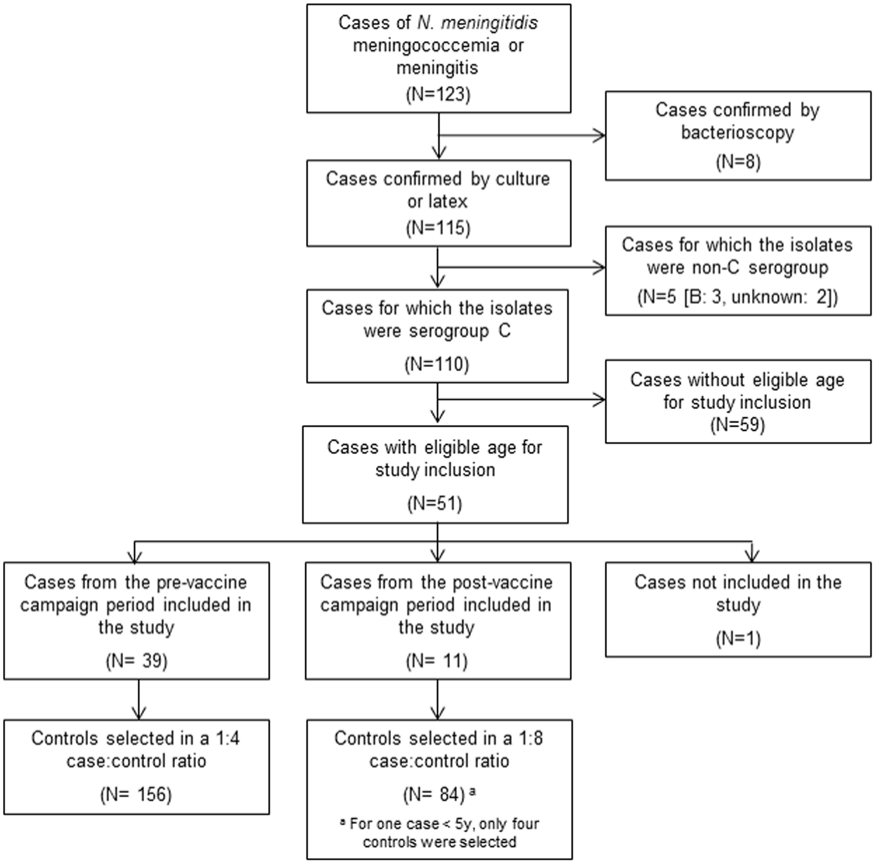
Design of case-control study. Of the 110 confirmed MenC cases, 88 (80%) were admitted to the public infectious diseases reference hospital (Couto Maia Hospital). Clinical presentations included meningococcemia (n = 43; 39%), meningitis (n = 32; 29%), and both meningococcemia and meningitis (n = 35; 32%) (Table 1). Reported symptoms included fever in all cases; headache, vomiting and other signs and symptoms of meningococcal infection were common. Diarrhea was reported in 11 (10%) of 110 cases. A total of 19 cases (17%) were admitted to an intensive care unit (ICU). Overall case-fatality was 25% (27 patients), including patients admitted to ICU and who died prior to ICU admission. Patients who died ranged in age from 0 to 68 years (median, 25 years). In addition, 11 (10%) patients recovered with neurologic sequella (motor deficit [n = 4], bilateral hearing loss [n = 2], visual impairment [n = 2] and lower limb amputation [n = 3]) (Table 1).

From January to December, 2010, 123 laboratory-confirmed cases of meningococcal disease were reported to the notifiable diseases reporting system in Salvador, Brazil (Fig 1). Serogroup was determined for 113 cases diagnosed by meningococcal culture or latex agglutination reaction; 110 (97%) were serogroup C and 3 were serogroup B. Serogroup was unknown for two cases confirmed by meningococcal culture and 8 cases diagnosed based on microscopic examination of CSF.

**Table 1:** Clinical characteristics of laboratory-confirmed cases of invasive meningococcal serogroup C disease, identified between 01/01/2010 and 31/12/2010 in Salvador, Brazil.

Characteristics	Age groups targeted for MenC vaccination	Age groups not targeted for MenC vaccination	Total (n = 110)
	(n = 51)	(n = 59)	
n (%) or mean ± SD			
**Age groups**			
0–5 years	6 (12)	-	6 (6)
6–9 years	-	11 (19)	11 (10)
10–14 years	14 (28)	-	14 (13)
15–19 years	13 (26)	-	13 (12)
20–24 years	17 (36)	-	17 (15)
> 25 years	-	48 (81)	48 (44)
**Male Sex**	32 (64)	35 (59)	67 (61)
**Clinical Presentation**			
Meningococemia	22 (43)	21 (36)	43 (39)
Meningitis	18 (35)	14 (24)	32 (29)
Meningococemia and meningitis	11 (22)	24 (40)	35 (32)
**Signs and Symptoms**			
Fever	51 (100)	59 (100)	110 (100)
Headache	41 (82)	52 (88)	93 (85)
Vomiting	36 (71)	44 (75)	80 (73)
Skin lesions	31 (62)	36 (61)	67 (61)
Neck stiffness	23 (46)	27 (46)	50 (45)
Abdominal pain	10 (20)	9 (15)	19 (17)
Lower limb pain	10 (20)	17 (29)	27 (24)
Seizures	7 (14)	3 (5)	10 (9)
Diarrhea	6 (12)	5 (8)	11 (10)
Altered mental status	5 (10)	19 (32)	24 (22)
**Laboratory**			
CSF^a^ white cells (mm^3^)	7.770±4.014	8.040±3.323	8.187±3.473
CSF protein (mg/dL)	387±228	408±215	397±214
CSF glucose (mg/dL)	29±17	28±14	28±15
White blood cells (X10^3/^mm^3^)	20±12	17±11	17±10
Platelets (X10^3/^mm^3^)	157±95	184±82	239±237
**Outcome**			
Death	12 (24)	15 (25)	27 (25)
ICU ^b^ admission	10 (20)	9 (15)	19 (17)
Permanent disabilities ^c^	05 (10)	06 (10)	11 (10)

^a^ CSF: Cerebrospinal fluid

^b^ ICU: Intensive Care Unit

^c^ Permanent disabilities included motor deficit (4), bilateral hearing loss (2), diplopia (2), lower limb amputation (3).

Of the 110 confirmed cases of meningococcal serogroup C disease reported in Salvador in 2010, 11 cases occurred among children aged 5–9 years and 48 cases occurred among adults aged 25 years or older, age groups not targeted for MenC vaccination and not included in the case-control study (Table 1). Of 51 MenC cases in 2010 aged <5 years or 10–24 years, 40 had symptom onset prior to MenC conjugate vaccine campaigns targeting their age group. One case in a two year old child from the pre-campaign period was excluded from the study because the case patient’s residence was not found and matched controls could not be enrolled. Among the 11 confirmed MenC cases in the post-campaign period, 4 occurred among children aged <5 years and 7 occurred among 10–24 year olds eligible to receive MenC vaccine in targeted city-wide vaccination campaigns.

A total of 50 confirmed cases of MenC disease were included in the case-control evaluation, including 12 (24%) of 27 fatal cases. Case patients were more likely to be male than age-matched neighborhood control individuals (64% vs. 44%, p<0.01; Table 2). Proportions of students among 10–24 year olds or working adults among those 18 years and older, household crowding and exposure to smoke were not statistically different between case and control individuals (Table 2). A total of 70 (30%) of the 240 control individuals received at least one dose of MenC vaccine at any time prior to enrollment in the study. Of these, 44 (63%) had documented record of vaccination date.

**Table 2:** Characteristics of case patients with laboratory-confirmed serogroup C meningococcal disease and age-matched residents of affected neighborhoods included in case-control study, Salvador, Brazil, 2010.

Characteristics	Cases (N = 50)	Controls (N = 240)
	N (%) or mean ±SD	
Age groups		
0–5 years	6 (12)	36 (15)
10–14 years	14 (28)	68 (28)
15–19 years	13 (26)	60 (25)
20–24 years	17 (36)	76 (32)
Male sex ^**a**^	32 (64)	106 (44)
Working, for those ≥ 18 years of age ^**b**^	6 (26)	26 (27)
Student, for those 10–24 years of age ^**c**^	27 (61)	111 (54)
Exposed to secondhand smoke at house	17 (34)	70 (29)
Number of residents per household	5.0±2.1	5.2±2.1
Ratio of number of residents at household per household room	1.2±0.6	1.2±0.6

^a^ Conditional logistic regression P<0.01. No other characteristic were statistically different between cases and controls.

^b^ Frequencies calculated for 23 cases and 98 controls older than 18 years, respectively.

^c^ Frequencies calculated for 44 cases and 204 controls with 10 to 24 years old, respectively.

Among 39 cases included from the pre-campaign period, none had received MenC conjugate vaccine, versus 11 (7%) of 156 age-matched neighborhood controls who received one or more doses of MenC conjugate vaccine at least 14 days before the corresponding case patient’s illness onset; of these, 9 received MenC conjugate vaccine in the private sector (Table 3). Exact odds ratio for vaccine effectiveness in the pre-campaign period was not statistically significant (Table 4). In the post-campaign period, 0 of 11 case patients and 59 (70%) of 84 matched control individuals had received MenC conjugate vaccine at least 14 days before the case patient’s symptom onset; 50 (85%) of 59 controls individuals received MenC vaccine in public vaccination campaigns. Combining both periods, overall effectiveness of MenC conjugate vaccines estimated from exact conditional logistic regression was 98% with a lower 95% confidence limit of 89% (Table 4).

**Table 3:** MenC vaccination status among control individuals before and after public MenC vaccination, according to age group and MenC vaccine provider.

Age group and vaccine source	MenC vaccination among control individuals before public vaccination campaign	MenC vaccination among control individuals after public vaccination campaign	P value^b^
	(N = 156) ^a^	(N = 84) ^a^	
	no. vaccinated / total (%)		
**Overall**	11/156 (7.1)	59/84 (70.2)	<0.001
**By age groups**			
0–5 years	1/8 (12.5)	20/28 (71.4)	0.005
10–14 years	2/44 (4.5)	19/24 (79.2)	<0.001
15–19 years	4/44 (9.1)	8/16 (50.0)	0.001
20–24 years	4/60 (6.7)	12/16 (75.0)	<0.001
**By vaccine provider**			
Private provider	9/156 (5.8)	9/84 (10.7)	0.130
Public provider	2/156 (1.3)	50/84 (59.5)	<0.001

^a^ Periods of MenC conjugate vaccine campaigns: February-December 2010 for <5 years old; May-August 2010 for 10–14 years old; June-August 2010 for 15–19 years old; and August 2010 for 20–24 years old.

^b^ Fisher exact test.

**Table 4:** MenC conjugate vaccine effectiveness and coverage among case and control subjects.

Subgroups	Cases (N = 50)	Controls (N = 240)	Odds Ratio (IC 95%) ^a^	Effectiveness in % (95% CI)	P value
	**Vaccine Coverage, n/N (%)**				
**Overall**	0/50 (0.0)	70/240 (29.2)	0.02 (0.00–0.10)	98.4 (89.8–100.0)	<0.001
**Overall by vaccination period** ^**b**^					
Before vaccine introduction	0/39 (0.0)	11/156 (7.1)	0.23 (0.00–1.51)	77.5 (0.0–100.0)^**c**^	0.142
After vaccine introduction	0/11 (0.0)	59/84 (70.2)	0.02 (0.00–0.11)	98.3 (88.6–100.0)	<0.001
**Only for those with vaccination status confirmed by immunization card verification** ^**d**^	0/33 (0.0)	44/103 (42.7)	0.03 (0.00–0.21)	97.0 (78.7–100.0)	<0.001

^a^ Exact odds ratio estimated using exact conditional logistic regression analysis.

^b^ Periods of MenC conjugate vaccine campaigns: February-December 2010 to <05 years old; May-August 2010 to 10–14 years old; June-August 2010 to 15–19 years old; and August 2010 to 20–24 years old.

^c^ The minimum level for the effectiveness lower confidence interval limit was set as zero.

^d^ Immunization card was available for verification for 33 of the 50 cases and for 103 of the 240 controls.

Among 11 case patients and 17 control individuals eligible for vaccination in campaigns, reasons for nonvaccination included lack of information about campaigns (7 [64%] of 11 cases and 6 [35%] of 17 controls), lack of time (4 cases and 2 controls) and not wanting to be vaccinated (9 controls)(Table 5).

**Table 5:** Reported reasons for non-vaccination among case patients with illness onset after public MenC vaccination and matched individuals from affected neighborhoods.

Reason for non-vaccination	Cases patients (n = 11) ^a^ ^,^ ^b^	Controls individuals (n = 17) ^b^
	n/N (%)	
Lack of time	4/11 (36)	2/17 (12)
Lack of information about the campaign	7/11 (64)	6/17 (35)
Did not want to be vaccinated	0/11 (0)	9/17 (53)

^a^ All 11 cases of *N*. *meningitidis* serogroup C invasive disease that occurred after MenC conjugate vaccine campaign initiation were unvaccinated.

^b^ Cases and controls frequencies for all the reported reasons for not being vaccinated after MenC conjugate vaccine campaign initiation were statistically different (Fisher exact P<0.01).

Of the 25 controls from the period after MenC conjugate vaccine campaign initiation, 8 had received the MenC conjugate vaccine within less than 14 days of occurrence of the matched case and, therefore, were not considered as vaccinated.

## Discussion

We performed the first case-control study in Brazil to evaluate effectiveness of MenC conjugate vaccine in containing a citywide epidemic of meningococcal disease. The epidemics were associated with the sequence types (ST) 3779 and 3780, both belonging to clonal complex 103 (Galvão, L et al. personal communication). This finding is of relevance as, to the present, effectiveness of MenC conjugate vaccines had only been shown against ST-11 meningococcal invasive strains. We also assessed vaccine uptake and identified reasons for non-vaccination. The results of this case-control study are consistent with a high effectiveness of meningococcal serogroup C conjugate vaccines, which have now been demonstrated in a variety of settings using different study designs [7,8,9]. Observational studies of MenC vaccine effectiveness are important because meningococcal conjugate vaccines were licensed based on evidence of an immune response in vaccinated subjects using serum bactericidal activity (SBA) as the immunologic correlate of protection rather than efficacy trials [10]. However, studies comparing trends in disease incidence before and after vaccine introduction may not provide accurate estimates of vaccine effectiveness because incidence of serogroup C meningococcal disease may vary greatly in the absence of vaccination, especially during localized epidemics [11].

Surveillance data from the city of Salvador showed declines in serogroup C meningococcal disease incidence that were greatest in age groups targeted for MenC vaccination [3]. Salvador was the only city in Brazil that conducted mass vaccination of teenagers in response to meningococcal C disease outbreaks. This strategy was adopted to reduce attack rates in heavily affected age groups as well as to reduce transmission by preventing meningococcal carriage among older children and adolescents. One dose of MenC conjugate vaccine has been shown to reduce carriage among older children [12,13]. This case-control study provided evidence that publicly-funded vaccination campaigns increased vaccine coverage in the targeted population residing in areas where meningococcal cases continued to occur. In addition, detailed vaccine histories collected during case investigations confirmed surveillance data that none of the patients with confirmed serogroup C meningococcal disease had received MenC vaccine prior to illness.

This case-control investigation was subject to several limitations. Active investigations for serogroup C meningococcal disease may have missed some cases as isolates were not obtained for all episodes of meningococcal disease for serogrouping. In addition, we cannot be certain that all recognized serogroup C meningococcal cases have been reported to the health department. Documentation of vaccination was not available for all individuals enrolled in the study and self-report may be biased; vaccination status was obtained by proxy for patients who died. Several studies have shown that exposure to cigarette smoke and household crowding are potential risk factors for meningococcal disease [14–17]. While exposures were similar among case-patients and control individuals, we could not investigate potential confounding of vaccine effects because none of the case-patients had received MenC vaccine. Finally, the study area was limited to the city of Salvador, Brazil, and did not include meningococcal cases or controls from other parts of the state of Bahia.

The United Kingdom was the first country to introduce meningococcal C conjugate vaccines and a number of vaccine effectiveness evaluations were conducted, including a case-control study that reported vaccine effectiveness of 93% (95% CI 39–99%) among adolescents aged 15–19 years [7]. Reductions in serogroup C meningococcal disease incidence following MenC vaccination and mass campaigns have since been reported from several settings [8,9,18]. Meningococcal serogroup C polysaccharide-protein conjugate vaccines were found to be safe and immunogenic in young children and were licensed in Brazil in 2003, but were only available in the public sector in Special Vaccine Reference Centers (*Centros de Referência para Imunobiológicos Especiais* [CRIES]) for children with high risk conditions or other indications (congenital or acquired asplenia, congenital immunodeficiencies, indication for cochlear implant, bone marrow transplant recipients and deposit [19]. MenC vaccines were introduced for universal infant vaccination in Brazil’s National Immunization Program in the second half of 2010 [20], but the state government of Bahia anticipated national introduction of routine childhood vaccination in response to the meningococcal epidemic to prevent cases in children. Following the initial introduction for children <5 years, serogroup C meningococcal disease continued to occur in older age groups, prompting mass vaccination of broader age groups. During the outbreak period, a total of 2,507 contacts of MenC cases were treated prophylactically with rifampin (n = 2,487) or ciprofloxicin (n = 20); no cases of meningococcal disease occurred among contacts.

Before publicly-funded MenC vaccination campaigns were conducted for targeted age groups, the epidemic of meningococcal disease in Salvador led to high demand for MenC vaccines in private clinics, where a vaccine dose cost approximately US$100. In the case-control study, few control individuals from areas where cases had occurred had purchased MenC vaccine in the private sector, while uptake of publicly-funded vaccine in targeted campaigns was much higher. The purchase of MenC vaccine by the state government of Bahia for mass vaccination of 10 to 24 year olds represented an investment of approximately US$ 30 million. The MenC vaccine was offered at no cost in city-wide campaigns targeted to age groups with the highest attack rates. Lack of information about these campaigns was one of the main reasons given for non-vaccination. Better communication strategies are needed to reach higher levels of vaccination coverage in emergency responses.

*Neisseria meningitidis* occasionally invades the bloodstream and cause serious invasive disease, manifesting as meningitis or septicemia [21–22]. High case-fatality and severity of meningococcal disease in affected individuals, including permanent sequelae, motivated the state government to conduct mass vaccination targeting the most affected age groups. In the present study, we observed that 71% of the cases progressed to septicemia and that 25% of them died. In Brazil, an analysis of clinical syndrome among meningococcal disease cases reported to Brazil’s national notifiable diseases system (SINAN) from 2000–2011 identified meningococcemia in 29%, meningitis in 39% and meningitis with meningococcemia in 32% [23]. Meningococcal disease particularly affects children aged <5 years, especially infants. However during outbreaks and epidemics, increased numbers of cases are often observed in adolescents and young adults [2,24]. During the epidemic of serogroup C meningococcal disease in Salvador, Brazil, adolescents experienced high attack rates.

## Conclusions

This case-control study provided critical information about vaccine uptake among targeted age groups in areas where meningococcal cases were occurring. Combating meningococcal disease epidemics in large populations is challenging. Mass immunization with MenC conjugate vaccine for target groups proved effective in preventing serogroup C meningococcal disease in Salvador, Brazil. MenC vaccination campaigns may be useful to prevent meningococcal C disease during epidemics.
